# PrEP in Italy: The time may be ripe but who's paying the bill? A nationwide survey on physicians' attitudes towards using antiretrovirals to prevent HIV infection

**DOI:** 10.1371/journal.pone.0181433

**Published:** 2017-07-20

**Authors:** Antonio Di Biagio, Niccolò Riccardi, Alessio Signori, Renato Maserati, Silvia Nozza, Andrea Gori, Stefano Bonora, Marco Borderi, Diego Ripamonti, Maria Cristina Rossi, Giancarlo Orofino, Tiziana Quirino, Giuseppe Nunnari, Benedetto Maurizio Celesia, Salvatore Martini, Caterina Sagnelli, Giovanni Mazzola, Pietro Colletti, Dario Bartolozzi, Teresa Bini, Nicoletta Ladisa, Filippo Castelnuovo, Annalisa Saracino, Sergio Lo Caputo

**Affiliations:** 1 Infectious Diseases Clinic, Department of Internal Medicine, Ospedale Policlinico San Martino, Genoa, Italy; 2 Department of Health Science, Biostatistics, University of Genoa, Genoa, Italy; 3 Malattie Infettive, Fondazione IRCCS Policlinico San Matteo di Pavia, Pavia, Italy; 4 Department of Infectious Diseases, IRCCS San Raffaele, Milan, Italy; 5 Clinic of Infectious Diseases, San Gerardo Hospital, University of Milano-Bicocca, Monza, Italy; 6 Unit of Infectious Diseases, Department of Medical Sciences, University of Torino, Torino, Italy; 7 Infection Diseases Unit, Sant’Orsola Hospital, University of Bologna, Bologna, Italy; 8 Infectious Diseases Unit, AO Papa Giovanni XXIII, Bergamo, Italy; 9 Unit of Infectious Diseases, Ospedale Regionale di Treviso, Treviso, Italy; 10 Unit of Infectious Diseases, "Divisione A", Ospedale Amedeo di Savoia, Torino, Italy; 11 Infectious Diseases Unit, Ospedali di Busto Arsizio, Varese, Italy; 12 Unit of Infectious Diseases, Department of Clinical and Experimental Medicine, University of Messina, Messina, Italy; 13 Department of Clinical and Molecular Biomedicine, Division of Infectious Diseases, University of Catania, ARNAS Garibaldi, Catania, Italy; 14 Department of Clinical and Experimental Medicine and Surgery "F. Magrassi e A. Lanzara, Second University of Naples, Naples, Italy; 15 Department of Medicinal Clinics and Emerging Diseases, "Paolo Giaccone" Polyclinic University Hospital, Palermo, Italy; 16 Infectious Disease Unit, Careggi University Hospital, Florence, Italy; 17 Clinical of Infectious Disease, San Paolo Hospital, Milan, Italy; 18 Institute of Infectious Disease, University of Bari, Bari, Italy; 19 Infectious Diseases, Spedali Civili Hospital, Brescia, Italy; University of New South Wales, AUSTRALIA

## Abstract

Several studies have demonstrated the efficacy of the oral pre-exposure prophylaxis (PrEP) with tenofovir (with or without emtricitabine) on preventing HIV-negative partners of HIV infected patients to become infected through sexual contacts. PrEP is already available in the United States and now is approved by European Medicine Agency. In this setting we would like to gauge physicians’ knowledge, acquaintance with and attitude to include PrEP in their clinical practice. A cross sectional survey was conducted among Italian physicians expert on antiretroviral therapy. Out of 146 physicians, 35% of participants declared to be familiar with PrEP but only 46% of them believed that, currently, there are not enough reasons to make it available in Italy. 51% of physicians have already been attracted to prescribe it and 63.4% have been openly asked about PrEP. The main concerns noticed were: the risk of acquire other sexual transmitted diseases (STDs) (70% of physicians feared that PrEP could favor STDs spread), the potential harmful of PrEP if not adequately implemented and, especially the risk of possible side effects if not properly used. Nevertheless, 55.9% of participants believed that Health Authorities face an ethical obligation to make PrEP available as part of the strategies to protect from HIV transmission and half of the respondents asked for further researches to better define the role for PrEP. Attitudes regarding PrEP impact on Italian National Health Organization were also very interesting: 57.5% of participants did not believe that investing in PrEP would be an appropriate use of healthcare resources, while 70.6% affirmed that PrEP’s financial coverage should not be funded by the Italian National System of Health (SSN). This survey showed a high awareness of PrEP potential among Italian physicians coupled with a great deal of skepticism about how and if implementing it in clinical practice.

## Introduction

Since the introduction of combined antiretroviral therapy (cART), mortality and morbidity rates among people living with HIV (PLWHIV) have constantly decreased. On the other hand, transmission is still ongoing, even though prevention tools and practices are widely available in developed countries [1,2]. Actually, significant efforts focused on information campaigns about condom use, and to promote access to therapies and counseling, the rate of new infections persists high and around 2 million people became infected in 2014 [3]. Plasma HIV-RNA reduction to below 50 copies/mL or very low levels by an effective cART treatment has already been demonstrated to be a valuable prevention strategy, thus reducing the sexual transmission in serodiscordant couples by a figure close to 90%[4–6]. Pre-Exposure Prophylaxis (PrEP) with oral tenofovir (TDF) alone, or in combination with emtricitabine (FTC), has been investigated in several randomized clinical (RCTs) trials enrolling men who have sex with men (MSM), heterosexuals, serodiscordant couples, and transgender women [7–13]. (Table 1)

**Table 1:** Completed trials of PrEP.

STUDY NAME	POPULATION	LOCATION	INTERVENTION	OVERAL REDUCTION IN HIV	PROPORTION WITH DETECTABLE DRUG IN BLOOD	RISK REDUCTION AMONG CONSISTENT USERS
**iPrEX**	MSM and transgender woman (n = 2499)	Brazil, Kenya, Peru, Ecuador, South Africa, Thailand, Uganda and US	Daily oral TDF/FTC	44% (95% CI: 15–63%)	51%	92% among participants with detectable drug in blood
**Partners in PrEP**	Heterosexual serodiscordant couples (n = 4578)	Kenya and Uganda	Daily oral TDF/FTC; daily oral TDF	TDF: 67% (95% CI: 44%-81%); TDF/FTC: 75% (95% CI: 55%-87%)	82%	86% (TDF) and 90% (TDF/FTC) among participants with detectable drug in blood
**TDF2**	Heterosexual men and woman (n = 1219)	Botswana	Daily oral TDF/FTC	62% (95% CI:22%-83%)	80%	78% among participants who refilled PrEP in the last 30 days
**Bangkok Tenofovir Study**	People who use injection drugs (n = 2413)	Thailand	Daily oral TDF	49% (95% CI: 10%-72%)	67%	70% among participants with detectable drug in blood
**FEM-Prep**	Women (n = 2120)	Kenya, South Africa and Tanzania	Daily oral TDF/FTC	6% (95% CI: -52% to 41%)	24%	NA
**VOICE**	Women (n = 5029)	South Africa, Uganda, Zimbabwe	Daily oral TDF, daily oral TDF/FTC, daily TDF vaginal gel	TDF: -49% (95% CI:-129% to 3%); TDF/FTC: -4% (95% CI: -49% to 27%); TDF gel: -15% (95% CI: -20% to 40%)	30%	66% among participants with detectable drug in blood (TDF gel arm)
**PROUD**	MSM (n = 545)	UK	TDF/FTC daily	86% (90% CI: 58%-96%)		
**IPERGAY**	MSM (n = 414)	France and Canada	TDF/FTC 2 to 24 hours before sex, followed by a third pill 24 hours after the first drug intake and a fourth pill 24 hours later	86% (95% CI, 40%- 98%)	86% for TDF and 82% for FTC in the first 113 patients enrolled	

The overall reduction in HIV risk transmission ranged from 44% to 86% and adherence to drugs emerged as the main factor associated with PrEP effectiveness. Actually the ability of PrEP to prevent HIV infection was consistently linked with detectable drug levels in blood, rising up to 74%-92% of risk reduction when occurring [14]. Other individual factors could limit PrEP success in real life, including drug-related safety issues, lack of awareness in the infected partners, risk compensation, etc. [15–17]. Most of these issues are currently investigated by open-label extension phase in some of the aforementioned studies. In July 2012, the American Food and Drug Administration has approved oral TDF plus FTC (TDF/FTC) for use as PrEP to reduce the risk of acquiring HIV infection in adults and recently the World Health Organization (WHO) and the Centers for Diseases Control (CDC) have included PrEP in their guidelines, recognizing the importance of recommending that Clinical and non-Clinical Providers should inform all HIV infected people, their HIV negative partners and intravenous drug users about the availability of PrEP [18–19]. At the time of this writing, the European Medicine Agency (EMA) has yet licensed TDF/FTC as PrEP in Europe, but the Italian Regulatory Agency has not yet approved it. However, two different European studies have shown the efficacy of PrEP as an addition to the current standards of prevention in MSMs [20–21]. The PROUD trial has investigated the impact of PrEP by comparing two groups of HIV-infected MSMs who had at least one unprotected episode of sexual exposure in the last 90 days. Participants were randomly assigned to receive a daily dose of TDF/FTC either immediately after the exposure or after a deferral period of 12 months. The results of the study showed that PrEP reduced the risk of HIV infection by 86% and was prematurely interrupted when an interim analysis revealed that 3 sero-conversions have occurred in the immediate group vs. 20 in the deferral group [20]. The IPERGAY study evaluated the efficacy of TDF/FTC vs placebo given not daily but at the time of sexual exposure in 414 MSMs and transgender women at high risk of acquiring HIV infection by an history of unprotected anal sex with at least two partners during the last 6 months. TDF/FTC or placebo were given with a loading dose of two pills 2 to 24 hours before sex, followed by a third pill 24 hours after the first drug intake and a fourth dose 24 hours later. The results of the study confirmed PrEP as effective, with only 2 HIV infections in the TDF/FTC group compared with 14 in the placebo group and a relative risk reduction of 86% (95%CI:40–98, p = 0.002) [18]. Because of these results, France approved PrEP in Nov 2015 [22]. Despite all these data, little is known about Clinicians willingness and doubts to consider PrEP in their practice at this time when access to PrEP is still limited or unavailable. Although PrEP was not yet part of guidelines prior to both of these studies, physicians demonstrated varying levels of support for PrEP and expressed concerns about its implementation [23–25].

Despite all these data, at the time of monitoring the PrEP was not authorized, the physicians demonstrate varying levels of support for PrEP and express concerns about its implementation with guidance from normative bodies. In Italy PLWHIV are looked after almost exclusively by Infectious Diseases Physicians who are also in charge of prescribing antiretrovirals. The entire combined antiretroviral treatment (cART) are only dispensed in Public Hospitals and are totally reimbursed by Italian National Health System (SSN), thus being free of charge for the patients. The latest epidemiological surveys done in Italy by the Istituto Superiore di Sanità showed that MSMs, sexually active heterosexual men and women are the persons most at risk of acquiring HIV [26]. Understanding the perceptions of Italian PLWHIV-caring physicians about PrEP and their readiness and doubts about it is important for a safe and effective PrEP prescription, when and if available in Italy too.

## Materials and methods

We conducted an anonymous survey by sending a questionnaire containing 21 items to 50 Italian Centers of Infectious Diseases, chosen as having a long-standing expertise on HIV treatment and distributed on the national territory. No informed consent was needed because the survey did not imply sensitive information or patients’ data. Besides, no ethics Committee approval was needed due to nature of collected data that were anonymous and were not possible to identify the participant physicians (S1 Table and S1 File). In Italy there are 153 HIV care centers, the median number of patients in follow-up is 624 with a range from 17 to 6,526 [27]; in our survey we involved physicians with more 500 patients in care. From April 1st, 2015 to May 30th, 2015, all physicians working in those selected centers, currently prescribing cART and likely to use PrEP when available, were invited to answer to the questionnaire. No patients were enrolled in this study and no ethics committee approval was needed. For all the physicians enrolled in the study, written informed consent was obtained. Both teaching (University) and non-teaching Hospitals were involved. No remuneration was provided for participants. The questionnaire was based on the Canadian experience recently published by Sharma et al. [5]. The questionnaire content was adjusted to fit the Italian healthcare and cultural scenario and was divided in three distinct areas as follows: I) three questions focused on the geographical area where the Physician is practicing, years of experience and practice-related details; II) seven questions regarding the attitudes, awareness and concerns with PrEP; III) eleven items about opinions on PrEP availability and its expected impact on National Health System. Areas of practice were clustered as North, Centre or South; the years of experience as a HIV/AIDS Physician were categorized in up to 5, 5 to 10, more than 15. Questions regarding the personal experience with PrEP were both YES/NO and multiples choice format and the questionnaire core part questions were answered through a 5 levels Likert Scale. Our main objective was to outline Clinicians opinion on PrEP availability in Italy (if there were currently enough reasons to authorize the availability of the PrEP; if they considered PrEP as an innovative tool for the prevention of HIV and if the drugs for PrEP should be provided free of charge by National Health System; if the clinicians were familiar with the PrEP; and if they have already been asked about PrEP by HIV-negative persons who believed to be at risk of acquiring HIV).

### Statistical methods

For the computation Stata (v.13; StataCorp.) was used. To statistically test association between questions in the three areas described above (ie: geographical differences or impact of personal professional experience on the PrEP availability’s opinion) Chi-square test or Fisher’s exact test, when appropriate, were used. Analysis on determinants of PrEP opinions was performed both on the original Likert scale and cumulating the scale in 3 levels (“Agree”, “Neutral” and “Disagree”) and this last one was reported. A p-value lower than 5% was considered statistically significant.

## Results

**Fig 1:**
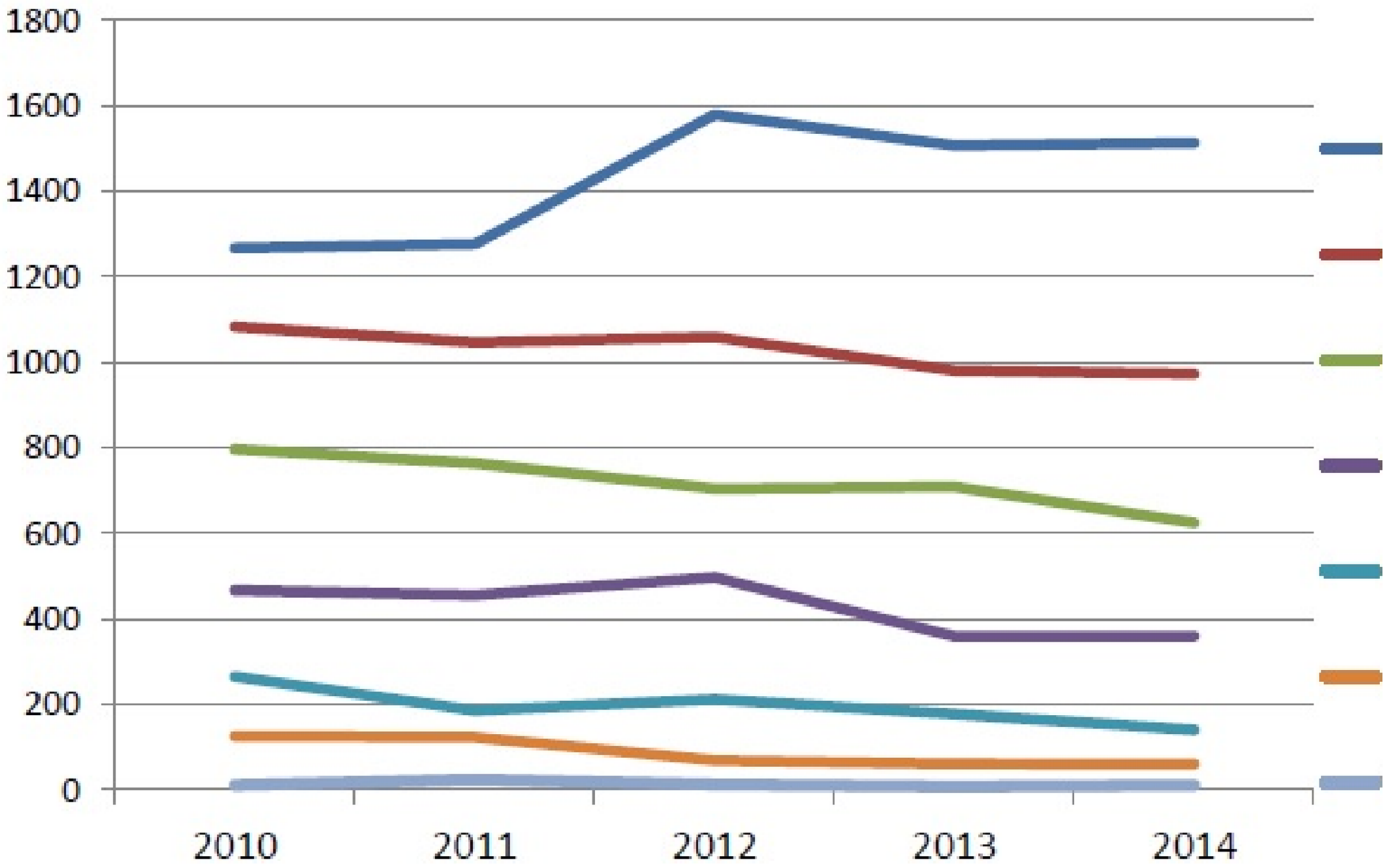
Number of new HIV diagnosis and mode of transmission of HIV in different years [26].

One hundred forty six questionnaires were received and included in the analysis. They were directly collected by local medical doctors and nobody refused to complete them. The geographical distribution was: 48.6% from Northern Italy, 23.3% from Central, and 28.1% from Southern Italy. Responding physicians confirmed to be highly experienced in taking care of PLWHIV and in the management of antiretroviral therapy, most of them being in that practice for more than 15 year (58.2%). The respondent’s professional activity was located in University (49.3%) or in other types of Hospital (50.7%). A significant difference between North, Central and South was observed when Physicians were asked about years of practice [p = 0.001; higher experience (15 years or more) in North and Central] and on location of their practice (p<0.001; more Physicians working in University Centers in Southern Italy). Physicians’ opinions on PrEP were investigated using a 5 levels Likert Scale, then condensed into three levels (Agree, Neutral, Disagree). Results of the 5 levels Likert Scale are shown in Fig 1.

For the question "Is there enough evidence to making PrEP available in Italy?" 46.9% of participants did not believe that there are enough reasons so far, 16.8% were undecided and only 36.3% believed that PrEP should be made available right away. For the question “PrEP could lead to medicalization of HIV prevention and shift focus from other objectives (condom, prevention campaign, etc.), 71.3% of respondents agreed or strongly agreed (41.8% strongly agreed, 29.5% agreed), somehow confirming concerns on risk-compensation. For the question “PrEP is an innovative HIV prevention tool and should be available as soon as possible 31.2% of participants considered PrEP innovative and asked for a fast local approval while 45.2% disagreed, 54.79% of them working in a non-teaching Hospital setting. 70.1% thought that this strategy could be dangerous if not used in an appropriate and safe way (31.9 strongly agreed; 38.2% agreed); physicians with a robust experience in HIV/AIDS should be in charge for this strategy of prevention as they already are for antiretroviral therapy (63%). 35.7% of respondent agreed when asked if “PrEP is mostly effective in men who have sex with men (MSM) but it is not so in other contexts”. All these concerns were expressed by 41.1% of participants who deemed PrEP not ready to be implemented and made widely available yet. This held true namely in Southern Italy where 48.7% of physicians did not believe it is time for such a strategy to be put in practice (p = 0.012). More than half of respondents (57.5%) did not believe that investing in PrEP would be an appropriate use of healthcare resources and 70.6% stated that PrEP’s financial coverage shouldn’t be afforded by SSN as it is for antiretroviral therapies. Nevertheless, 55.9% of participants were of the opinion that the Health Authorities have an ethical obligation to make PrEP available as part of the strategies to curb HIV transmission; this last expression of views was shared by 63.4% of respondents practicing in a University setting vs. the 46.6% only of non-teaching Hospitals Physicians. Out of 146 physicians answering the question "Are you familiar with PrEP?" 65.0% responded “NO”, although 61.2% of them declared to be expert in the field of HIV/AIDS and having been in that practice for more than 15 years. Among respondents working in Southern regions, “YES” answer to this question was higher (65%) than “NO” (35%), being a negative reply prevalent in Northern and Center respondents (78.57% and 72.73%, respectively). At the question: Are you tempted to prescribe PrEP? 51.4% of physician reply YES. Italian HIV/AIDS physician did not consider PrEP useful for the MSM community only but a key strategy for other groups as serodiscordant couples (50.68%).

## Discussion

Our first objective was to assess Clinician’s perception about PrEP availability in Italy seemed to reflect a quite prudent approach. A first barrier to PrEP adoption seemed to be the potential, relative decline of other types of prevention that is confirmed by the answers. A lack of innovative profile perception emerged when asked to judge the statement of innovation of the approach. Despite different opinions on its availability, most of the participants in this survey agreed on the harmful potential of PrEP if not adequately implemented and most of them thought that this strategy could be dangerous if not used in an appropriate and safe way. There was also a broad consensus about the identification of individuals at risk and the offer PrEP remains crucial for its success. Another limit to PrEP acceptance may be the perception that it may work only for a restricted group of individuals at very high risk. Answers regarding the potential impact of PrEP on SSN resources were also very interesting. Exploring the personal knowledge of PrEP among Italian Physicians may be crucial for understanding their willingness to prescribe it in the next future. Overall, a very conservative approach was shown by Italian physicians: in fact when asked about PrEP the large majority of very experienced respondents declared to be neutral or not to have a clear belief about it. Despite differences regarding the physician population and the Health System organization compared to those of countries where PrEP was studied or authorized, the results of the research provide useful information about PreP attitudes in Italy. When comparing to a similar study conducted in Canada [25], the number of Physicians interviewed was higher (146 vs 104) and they were all Infectious Disease Specialists with a fairly significant experience in the HIV/AIDS field. However, the main limitation of our study remains the number of questionnaires delivered (146) on approximately 200 Physicians who currently visit patients living with HIV in Italy. Our experience showed that Italian HIV Physicians are less familiar with PrEP than their Canadian counterpart, at the same time they are more attracted to prescribe PrEP. Furthermore our study was performed after the results of the RCTs demonstrating the efficacy of PrEP [20–21]. If we consider that currently the pharmaceutical company manufacturing the compounds so far used in this setting does not promote in any way PrEP in Italy and that the survey has been conducted before the WHO recommendation (18), the level of insight into this prevention strategy resulted to be by far better than expected. It would be interesting to conduct further analysis if we assume that the positive results of both studies could have an impact on Physicians opinion on PrEP. In facts, in our analysis, familiarity is the main factor associated to the willingness of availability and 48% of physicians declaring familiarity to PrEP, do not believe that there are not enough reasons to make it available (p = 0.005). Moreover, familiarity is also related to temptation to prescribe, as shown by the Southern respondents.

Awareness is crucial for preventing HIV transmission: physicians are essential for patient training and information about a correct use of PrEP and they should be supported with educational tools to fully understand all advantages and limits before PrEP become widely available. Half of the respondents ask for further researches, which could better define the role for PrEP in a real life setting and in different populations. These interesting results could represent the beginning of a dialogue with Health Authorities about its availability. Nearly two-thirds of physicians fear a lowering of the attention towards all sexually transmitted diseases, in agreement with Canadian paper colleagues, where over 60% of physician be afraid that PrEP could lead the medicalization of HIV prevention and shift focus from other efforts. Answers regarding impact on SSN resources were also very interesting: almost of 60% respondents don’t believe that investing in PrEP would be an appropriate use of healthcare resources and 70.6% affirm that PrEP’s financial coverage shouldn’t be afforded by SSN as for antiretroviral therapies. In Italy the budget of antiretrovirals drugs is managed by the single department of infectious diseases inside a Hospital budget. Portfolio includes all other items of expenditure (i.e. diagnostics, other drugs, staff salaries). The current economic recession has led some Italian Hospital to adopt austerity polices in order to control the prescription of antiretroviral therapies, thus expense is a major concern for Italian Physicians.

In conclusion, this survey, at the best of our knowledge the first in Italy and in Europe, showed a high awareness of PrEP potential among Italian physicians coupled with a great deal of skepticism on how and if implementing it in clinical practice. Main concerns are the risk that PrEP could favor STDs spread, the potential harmful of PrEP if not adequately implemented and by and large the dangers if not used in an appropriate and safe way. Participants believed that SSN face an ethical obligation to make PrEP available as part of the strategies to protect from HIV transmission and half of the respondents asked for further researches to better define the role for PrEP.

## Supporting information

S1 Table (DOCX)

S1 File Database. (XLSX)
