# Supplementary material for: PrEP in Italy: The time may be ripe but who's paying the bill? A nationwide survey on physicians' attitudes towards using antiretrovirals to prevent HIV infection
Source: PLoS One. 2017 Jul 20;12(7):e0181433. doi: 10.1371/journal.pone.0181433 (PMC5519091; doi:10.1371/journal.pone.0181433)
Supplement: S1 Table — (DOCX) [file pone.0181433.s001.docx]

S1 Table

What do you think about PrEP ?

**Introduce Yourself**

1. Where do you work?
2. North of Italy
3. Centre of Italy
4. South of Italy
5. For How long have you been treating HIV-infected patients?
6. 5 years
7. 10 years
8. over 15 years
9. My workplace is:
10. University Hospital
11. General Hospital

**Questions**

 You can depict their level of agreement according to the following rating scale

| ***Strongly agree*** | ***Agree*** | ***Neutral*** | ***Disagree*** | ***Strongly disagree*** |
| --- | --- | --- | --- | --- |
| ***1*** | ***2*** | ***3*** | ***4*** | ***5*** |

1. In 2015, there were not enough reason to make PrEP available in Italy?

| ***1*** | ***2*** | ***3*** | ***4*** | ***5*** |
| --- | --- | --- | --- | --- |

1. Invest in PrEP do you think is an appropriate use of Sistema Sanitario Nazionale (SSN) resources?

| ***1*** | ***2*** | ***3*** | ***4*** | ***5*** |
| --- | --- | --- | --- | --- |

1. PrEP should be provided free by the SSN?

| ***1*** | ***2*** | ***3*** | ***4*** | ***5*** |
| --- | --- | --- | --- | --- |

1. PrEP should be prescribed by Infectious Diseases specialist and distributed in pharmacies?

| ***1*** | ***2*** | ***3*** | ***4*** | ***5*** |
| --- | --- | --- | --- | --- |

1. Decision makers (Institution of Italy) have an ethical obligation in respect of PrEP or otherwise of each intervention to protect people from an infection like HIV

| ***1*** | ***2*** | ***3*** | ***4*** | ***5*** |
| --- | --- | --- | --- | --- |

1. PrEP could lead to the medicalization of HIV prevention and shift focus to other targets (condoms, awareness campaigns, syringes, etc)?

| ***1*** | ***2*** | ***3*** | ***4*** | ***5*** |
| --- | --- | --- | --- | --- |

1. PrEP is an innovative tool for the prevention of HIV and should be made available as soon as possible?

| ***1*** | ***2*** | ***3*** | ***4*** | ***5*** |
| --- | --- | --- | --- | --- |

1. PrEP could do more harm than good, if not properly used?

| ***1*** | ***2*** | ***3*** | ***4*** | ***5*** |
| --- | --- | --- | --- | --- |

1. PrEP can be useful, but is not yet time to be widely used?

| ***1*** | ***2*** | ***3*** | ***4*** | ***5*** |
| --- | --- | --- | --- | --- |

1. PrEP is supported only by the gay community and is not effective in other contexts?

| ***1*** | ***2*** | ***3*** | ***4*** | ***5*** |
| --- | --- | --- | --- | --- |

1. PrEP is dangerous and should not be pursued further?

| ***1*** | ***2*** | ***3*** | ***4*** | ***5*** |
| --- | --- | --- | --- | --- |

1. Which of these factors you consider important to eventually implement PrEP?
2. Efficacy
3. Availability of PrEP in Italy
4. Prescription control or PrEP Stewardhisp
5. Cost-effectiveness
6. Side Effects
7. Adherence
8. Resistance
9. Taking responsibility for decision makers (SSN, Regioni, Scientific Society)

*Put in order of preference*

| 1 |  |
| --- | --- |
| 2 |  |
| 3 |  |
| 4 |  |
| 5 |  |
| 6 |  |
| 7 |  |
| 8 |  |

**Questions about Your experience**

1. You are comfortable with PrEP?
2. Yes
3. NO
4. Some people not infected with HIV asked you what is PrEP?
5. Yes
6. NO
7. If yes, WHO?
8. Sero-discordant couple
9. Sex workers
10. Intravenous Drug Abuser
11. Have you ever been tempted to prescribe PrEP?
12. Yes
13. NO
14. If Yes, to who?
15. Sero-discordant couple
16. Sex workers
17. Intravenous Drug Abuser
18. What is your attitude when you are asked about PrEP by patients or partners of patients?
19. Enthusiast
20. Neutral
21. Disillusioned
22. Realistically in 2020 in Italy will PrEP be available?
23. Yes
24. NO
